# Angiotensin I-Converting Enzyme Gln1069Arg Mutation Impairs Trafficking to the Cell Surface Resulting in Selective Denaturation of the C-Domain

**DOI:** 10.1371/journal.pone.0010438

**Published:** 2010-05-03

**Authors:** Sergei M. Danilov, Sergey Kalinin, Zhenlong Chen, Elena I. Vinokour, Andrew B. Nesterovitch, David E. Schwartz, Olivier Gribouval, Marie-Claire Gubler, Richard D. Minshall

**Affiliations:** 1 Department of Anesthesiology, University of Illinois at Chicago, Chicago, Illinois, United States of America; 2 Department of Pharmacology, University of Illinois at Chicago, Chicago, Illinois, United States of America; 3 Hôpital Necker-Enfants Malades, INSERM, U574, Université Paris Descartes, Paris, France; 4 National Cardiology Research Center, Moscow, Russia; Roswell Park Cancer Institute, United States of America

## Abstract

**Background:**

Angiotensin-converting enzyme (ACE; Kininase II; CD143) hydrolyzes small peptides such as angiotensin I, bradykinin, substance P, LH-RH and several others and thus plays a key role in blood pressure regulation and vascular remodeling. Complete absence of ACE in humans leads to renal tubular dysgenesis (RTD), a severe disorder of renal tubule development characterized by persistent fetal anuria and perinatal death.

**Methodology/Principal Findings:**

Patient with RTD in Lisbon, Portugal, maintained by peritoneal dialysis since birth, was found to have a homozygous substitution of Arg for Glu at position 1069 in the C-terminal domain of ACE (Q1069R) resulting in absence of plasma ACE activity; both parents and a brother who are heterozygous carriers of this mutation had exactly half-normal plasma ACE activity compared to healthy individuals. We hypothesized that the Q1069R substitution impaired ACE trafficking to the cell surface and led to accumulation of catalytically inactive ACE in the cell cytoplasm. CHO cells expressing wild-type (WT) vs. Q1069R-ACE demonstrated the mutant accumulates intracellularly and also that it is significantly degraded by intracellular proteases. Q1069R-ACE retained catalytic and immunological characteristics of WT-ACE N domain whereas it had 10–20% of the nativity of the WT-ACE C domain. A combination of chemical (sodium butyrate) or pharmacological (ACE inhibitor) chaperones with proteasome inhibitors (MG 132 or bortezomib) significantly restored trafficking of Q1069R-ACE to the cell surface and increased ACE activity in the cell culture media 4-fold.

**Conclusions/Significance:**

Homozygous Q1069R substitution results in an ACE trafficking and processing defect which can be rescued, at least in cell culture, by a combination of chaperones and proteasome inhibitors. Further studies are required to determine whether similar treatment of individuals with this ACE mutation would provide therapeutic benefits such as concentration of primary urine.

## Introduction

Angiotensin I-converting enzyme (ACE, CD143) is a Zn^2+^ carboxydipeptidase which plays a key role in the regulation of blood pressure and also in the development of vascular pathologies and tissue remodeling [1]–[4]. ACE is expressed as two isoforms: somatic ACE (sACE), which is responsible for its hypertensive properties, and a smaller isoform (testicular ACE), which is expressed solely in germinal cells. sACE is highly expressed in endothelial [5]–[6], epithelial, neuroepithelial [7]–[8], and immune cells (macrophages and dendritic cells) [9]–[10] as a membrane-bound protein and has recently been designated as CD143 [11]–[12]. Two homologous domains (N and C domains) comprise the majority of the structure of sACE, each containing a functional active center [13]. The three-dimensional structure of sACE is currently unknown, but some indication of the interaction of the two domains has been inferred from *in silico* analysis [14]–[16] of the recently solved crystal structures of the N and C domains [14], [17].

Numerous data convincingly indicate that elevated ACE expression is a risk factor associated with several cardiovascular and renal diseases such as hypertension, cardiac hypertrophy, diabetic nephropathy, and others [18]–[19]. Deficiency in ACE due to ACE inhibition or complete absence of ACE due to genetic manipulation or mutations also leads to severe disease phenotypes including defects in fetal development, hypotension, inability to concentrate urine, structural renal defects, anemia, and reduced male fertility [4], [20]. In large mammals (rabbit, sheep, baboon), ACE deficiency results in low birth weight, preterm delivery, and fetal death [21]. In the same manner, human fetuses exposed to ACE inhibitors during the second and third trimesters of gestation are at risk of developing a fetopathy characterized by anuria-oligohydramnios, hypotension, growth restriction, renal tubular dysgenesis and hypocalvaria [22]. More recently, increase risk for congenital malformations of the cardiovascular and the central nervous systems has been reported in infants with first trimester exposure to the drug [21], [23]. Therefore, up-regulation of ACE expression may be an important therapeutic strategy for patients with low ACE levels.

Two mutations in ACE have been described which were linked to premature fetal death due to autosomal recessive renal tubular dysgenesis -RTD [24]–[25], a severe disorder of renal tubular development characterized by persistent fetal anuria and perinatal death, probably due to pulmonary hypoplasia from early-onset oligohydramnios (Potter phenotype). Affected individuals die *in utero* or within 24 hr of birth. RTD is genetically heterogeneous and linked to mutations in genes that encode components of the renin-angiotensin system. In the two cases described, the etiology of RTD was linked to mutations in ACE that lead to non-functional ACE protein in the homozygous state, a frame shift mutation in the 8^th^ exon or introduction of a stop codon in the 5^th^ exon, both of which resulted in a truncated protein the lacked the catalytic sites [24].

Herein, we report a mechanism for the 3^rd^ case of RTD which was observed in Lisbon, Portugal which is associated with complete absence of ACE due to a homozygous Q1069R ACE substitution. We present evidence that this novel mutation represents a “***transport –defective*** or -***processing***” mutant of ACE. Moreover, we demonstrate in a CHO cell expression model that a combination of chemical (sodium butyrate) or pharmacological (ACE inhibitor) chaperones with proteasome inhibitors (MG 132 or bortezomib) significantly restored trafficking of mutant ACE to the cell surface. Therefore, chaperones and proteasome inhibitors may be clinically useful for restoring cell surface expression of “***transport –defective*** or -***processing***” mutant proteins.

## Materials and Methods

### DNA preparation, linkage analysis and mutation screening

Peripheral blood was obtained from the affected patient, her unaffected brother, and her parents by Dr. Rosario Stone (Hospital de Santa Maria, Lisboa, Portugal) after obtaining informed consent from parents. Blood samples were sent to Hôpital Necker-Enfants Malades, INSERM, U574, Université Paris Descartes, Paris, France. Experiments were done in accordance with French ethical committee recommendation. Genomic DNA was isolated by standard methods. Linkage analyses were performed using microsatellite markers based on proximity to *REN, AGT, AGTR1* and *ACE* genes as well as marker heterozygosity (D1S2683, D1S2717, D1S2668, D1S2872 for *REN*; D1S479, D1S2833, D1S225, D1S251 for *AGT*; D3S3618, D3S1306, D3S1308, D3S3705 for *AGTR1*; D17S948, D17S944, D17S1809 and D17S1874 for *ACE*). Linkage to the *ACE* locus was compatible whereas the genes *REN*, *AGT* and *AGTR1* could be excluded as the cause of the disease.

We used the Oligo 6.2 program (NBI) to design specific primers to amplify the coding exons and the adjacent intronic sequences of the *ACE* gene (26 exons). The PCR products were treated with Exo-SAP IT (AP Biotech) and both strands were sequenced using the dideoxy chain termination method on a 3130XL DNA sequencer (Applied Biosystems). Sequences were evaluated with the Sequencer software (Gene Codes, Ann Arbor, MI, USA). Segregation analysis of mutations in families and 100 unrelated ethnically matched controls was performed by direct sequencing. Amino acid conservation at the missense mutations was assessed using SIFT software (http://sift.jcvi.org/) and Polyphen was used to predict the effect of the variation on the protein (http://coot.embl.de/PolyPhen/).

### ACE activity assay

Heparinized plasma from patient, her parents and healthy brother, kindly provided by Dr. Rosario Stone (Hospital de Santa Maria, Lisboa, Portugal), was taken for determination of ACE activity. The study was approved by the Institutional Review Boards of the University of Illinois at Chicago and procedures followed were in accordance with institutional guidelines. ACE activity in human plasma was measured using a fluorimetric assay [26]–[27]. Briefly, 20–40 µl aliquots of heparinized plasma, diluted 1/5 in PBS + BSA (0.1 mg/ml), were added to 200 µl of ACE substrate (5 mM Hip-His-Leu or 2 mM Z-Phe-His-Leu) and incubated at 37^°^C. The His-Leu product was quantified by incubation with O-phthaldialdehyde fluorimetrically at 365 nm excitation and 500 nm emission wavelengths. Determination of the ratio of hydrolysis of the two substrates (ZPHL/HHL) was performed as described [28].

### Site-directed mutagenesis and *in vitro* analysis of mutant ACE

cDNA encoding mutant ACE protein was provided by Dr. Tiago Outeiro (Cell and Molecular Neuroscience Unit, Instituto de Medicina Molecular, Lisboa, Portugal) and was created by mutation of the CAG codon for Gln at position 1069 to codon CGG for Arg in pACE-wt, an expression vector based on pcDNA3.1+/Hygro (Invitrogen Corp., Carlsbad, CA) (provided by us), containing the full-length somatic ACE cDNA controlled by CMV early promoter [29]. The exact procedure will be described and published elsewhere. Plasmids carrying the coding sequence for wild-type (WT) ACE and mutant ACE (Q1069R) were expressed in CHO cells using Plus Reagent (Invitrogen Corp., Carlsbad, CA) for transient transfection and generation of stable cell lines was performed by selection in G418 containing media according to manufacturer's recommendation. CHO cell lines expressing human recombinant wild-type have been previously described [29].

Culture medium (Ultra-CHO medium, Cambrex Bio-Science Walkersville, Inc, Walkersville, MD) from these cells (WT-ACE and Q1069R) was used as a source of the soluble ACEs, and cell lysates as a source of membrane-bound ACEs for biochemical and immunological characterization. To assess the effect of culture conditions and different compounds on ACE activity and localization, CHO cells stably expressing WT- and Q1069R-ACE were cultured in F12 medium containing 10% Fetal Bovine Serum (FBS). When the cells reached 70–90% confluence they were washed twice with PBS and fresh Ultra-CHO medium having no endogenous ACE activity was added. Effect of temperature (30°C versus 37°C) and inhibitors was assessed: sodium butyrate (Sigma-Aldrich, St. Louis, MO)-5 mM; ACE inhibitor enalaprilat (Sigma-Aldrich, St. Louis, MO)-1 µM; proteosome inhibitors–MG132 (Assay Designs, Ann Arbor, MI) and Bortezomib (ChemieTek, Indianapolis, IN)-both at 5 µM. After 24 hrs, the culture medium was aspirated and centrifuged (to removed detached cells) and the cells, after washing with PBS, were lysed with 50 mM Tris-HCl buffer, pH 7.5, containing 150 mM NaCl and 0.5% Triton X-100. ACE activity in the lysate and culture medium was determined using two ACE substrates as described above.

### Western blot analysis of mutant ACE expression

Lysates from CHO-WT-ACE cells (with ACE activity of 8 mU/ml using Hip-His-Leu) were compared to lysates from CHO-ACE–Q1069R cells normalized by equal protein loading by SDS-PAGE in 4–15% acrylamide Tris-HCl pre-cast SDS PAGE gels (Bio-Red Laboratories, Hercules, CA). After electrophoretic transfer of proteins to microporous PVDF-Plus membranes, each membrane was incubated 30 min in 10 mM Tris-HCl (pH 8.0) buffer containing 150 mM NaCl, 0.05% Tween 20, and Western Blocking solution (Sigma-Aldrich, St. Louis, MO) prior to incubation with hybridoma culture fluids (1/10 dilution in the same blocking solution) for 1 hr at room temperature. Subsequent steps were carried with secondary antibodies (ant-mouse and anti rat IgG, depending on primary antibody to ACE, conjugated with peroxidase) and peroxidase activity was developed using SuperSignal WestPico Chemiluminescense substrate (Pierce, Rockford, IL).

### mRNA quantification

Total RNA was prepared from stably transfected CHO cells (CHO-WT-ACE and CHO-Q1069R-ACE) using TRIZOL reagent (Invitrogen Corp., Carlsbad, CA) and treated with DNase (Ambion., Austin, TX) to remove DNA. RNA was converted to cDNA by SuperScript® III Reverse Transcriptase (Invitrogen Corp., Carlsbad, CA) using random hexamer primers, and mRNA levels were estimated by quantitative touchdown PCR (QPCR). PCR was done using Taq DNA Polymerase (Invitrogen Corp., Carlsbad, CA) and contained SYBR Green (SybrGreen1 10,000× concentrate, diluted 1∶10,000; Molecular Probes, Eugene, OR). PCR conditions were 30 cycles of denaturation at 94°C for 10 s; annealing at 56–62°C for 15 s; and extension at 72°C for 20 s on a Corbett Rotorgene Real-Time PCR unit (Corbett, Australia). Relative mRNA concentrations were calculated from the takeoff point of reactions using manufacturer's software and normalized to α-tubulin. Melting curve analysis and agarose gel electrophoresis ensured production of single and correct-size products. The primers used were:

α-tubulin forward, CCCTCGCCATGGTAAATACAT;

α-tubulin reverse, ACTGGATGGTACGCTTGGTCT;

ACE forward, TCCGCACGGAGAACGA

ACE reverse, CCTGCTGCGCATCCA

### Confocal microscopy

To assess the cell surface and intracellular localization of exogenously expressed WT- and Q1069R-ACE, CHO cells were either fixed in 0.3% paraformaldehyde on ice for 15 min, or fixed and permeabilized with 3% paraformaldehyde at 37°C for 15 min, respectively. Fluorescent secondary Ab (goat anti-mouse Alexa 488, 1∶500) labeling of anti-ACE mAb 9B9 [30] was used to label and localize WT and Q1069R ACE and nuclei were labeled with DAPI (1 µg/ml). Traditional line scanning confocal microscopy [31] using a Zeiss LSM 510 META microscope, 488 nm laser excitation/520 nm emission for Alexa 488 fluorescence and Hg lamp + UV filter set for DAPI, 63x 1.2 NA water immersion objective, and pinhole set to achieve 1 Airy unit was used to image the expressed ACE constructs in CHO cells.

### Immunological characterization of the mutant ACE (Plate immunoprecipitation assay)

96-well plates (Corning, Corning, NY) were coated with 50 µl affinity-purified goat anti-mouse IgG (Pierce, Rockford, IL) at 10 µg/ml and stored overnight at 4°C. After washing with PBS containing 0.05% Tween 20, the wells were incubated with different anti-ACE mAbs (2 µg/ml) directed to 16 different epitopes on the N and C domain of ACE [15]–[16], [30], [32]–[34] in PBS + 0.1 mg/ml BSA for 2 hrs at RT and washed. Wells were then incubated with 50 µl of soluble ACE secreted from CHO cells transfected with wild type or mutant ACE or with lysates from these cells, resembling a membrane-bound form of ACE (wt and mutant). These samples (wt versus mutant ACE) were equilibrated for determination of ACE activity using Z-Phe-His-Leu as a substrate for 2 hrs at RT. After washing away unbound ACE, plate-bound ACE activity was measured by adding the ACE substrate (Hip-His-Leu or Z-Phe-His-Leu) directly into wells [30].

## Results and Discussion

### Identification of ACE mutation

Diagnosis of Renal Tubular Dysgenesis (RTD) was made in the first day of life of the patient by Dr. Rosario Stone (Hospital de Santa Maria, Lisboa, Portugal) based on hypocalvaria, hypotension, and anuric renal failure. Clinical characterization of this case will be published elsewhere. Based on the results of Gribouval et al. [24], it was proposed that the homozygous lost-of-function mutation in one of the genes responsible for AII-dependent signaling – angiotensinogen (AGT), renin (REN), angiotensin-converting enzyme (ACE), or the receptor for angiotensin II (AGTRI), might be responsible for the phenotype. Genomic DNA from this patient, her parents and healthy brother was sent to Dr. M-C. Gubler (Hôpital Necker-Enfants Malades, INSERM, U574, Université Paris Descartes, Paris, France).

Sequencing of the 26 exons of the *ACE* gene led to the identification in exon 22 of the c.3293A>G (p.Q1098R) mutation in the homozygous state in the patient and the heterozygous state in each parent and the patient brother. In mature somatic ACE protein (without signal peptide-mature somatic ACE numbering [13] it correspond to Q1089R. This mutation was found in both parents and in the brother of the patient in the heterozygous state. According to database search (http://www.ncbi.nlm.nih.gov/projects/SNP/), there is no variant in *ACE* exon 22 and screening results revealed that none of the 100 healthy controls showed this mutation indicating that this substitution is not a polymorphism. This mutation results in substitution of a highly conserved glutamine with lysine in position 1069 in the C-terminal domain of ACE. The amino acid change is not tolerated according to SIFT software and Polyphen predicts this mutation is probably damaging with a score at 1.868.

### ACE activity determination in the affected family

**Figure 1:**
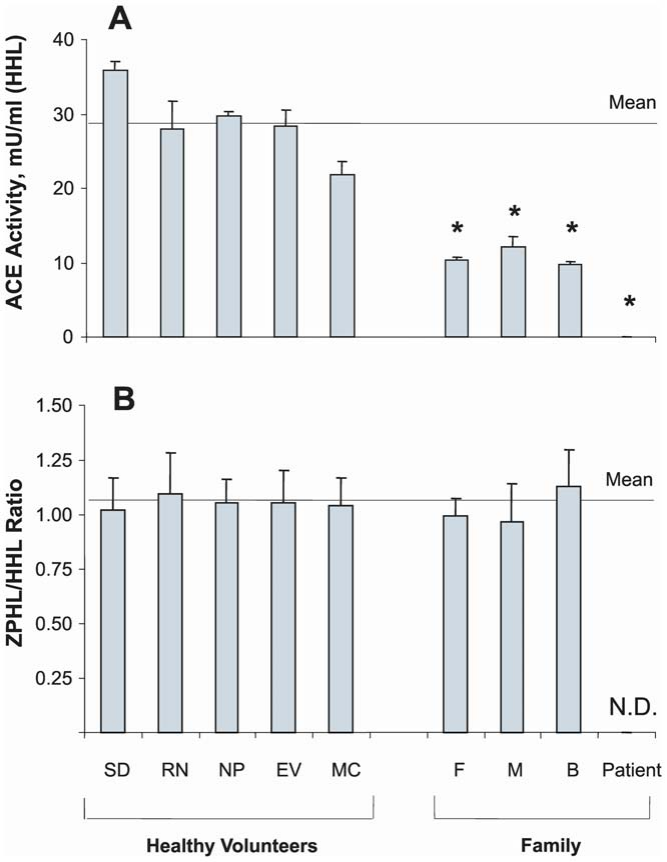
Plasma ACE activity of the members of the affected family. ACE activity in heparinized plasma (1/5 dilution in PBS) of members of the affected family, the father (F), mother (M), and brother (B) as well as in 5 healthy individuals (marked by initials) that served as controls was determined by fluorimetric assay of 40 µl diluted plasma with 200 µl each of substrates Hip-His-Leu (HHL) and Z-Phe-His-Leu(ZPHL) during 1 hour of incubation. **A.** ACE activity with HHL: mean (± SD) of three independent determinations. **B.** Ratio of hydrolysis of the two substrates (**ZPHL/HHL ratio**) in the tested samples, which is used to characterize for each catalytic domain of ACE in somatic two-domain ACE [28]. ND-not determined. * p<0.05 vs. control samples.

**Figure 2:**
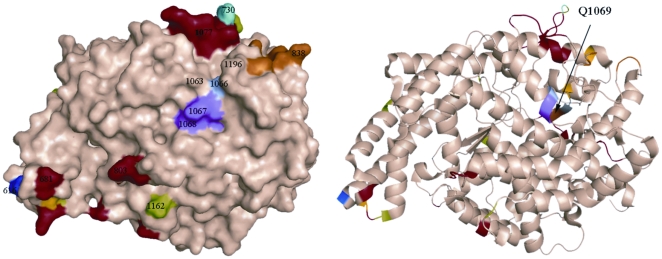
Localization of new mutation (Q1069R) in the C domain of somatic ACE. The localization of Gln1069 in the C-domain of human ACE which was mutated to Arg is shown using molecular surface (**A**) and ribbon (**B**) representations of the substrate-bound crystal structure of the C-domain fragment of human ACE, where the 36 amino acid residues unique to tACE were deleted [75]. The surface and ribbon are light brown, and amino acid residues that were crucial for orientation are colored. Key amino acids referred to in the text are denoted using somatic ACE numbering. Gln1069 (brown) is located right under the bump created by Lys1067 and Tyr1068 (purple) and shown by arrow. Dark blue indicates the first N-terminal amino acid residue (Asp616) seen in this structure; orange-indicates the C terminal end of the C-domain (and the epitope for mAb 1B3; [16], [36]. Light blue (P730) and red colored amino acid residues indicate highly immunogenic bumps on the surface of the C-domain. Light green indicates Asp in putative glycosylation sites.

Plasma ACE activity of the patient and immediate family were measured and showed that the patient had no ACE activity whereas both parents and a brother had approximately two-times less ACE compared to healthy individuals (Figure 1). Figure 1A shows ACE activity (cleavage of substrate Hip-His-Leu) in heparinized plasma of 5 healthy individuals and the 4 members of the affected family. Figure 1B, which shows the ratio of the rate of hydrolysis of two ACE substrates, Z-Phe-His-Leu and Hip-Hi-Leu (ZPHL/HHL ratio, used to characterize the N and C domains of ACE and thereby assess the nativity of somatic ACE [28], was not different in the parents and brother compared to ACE activity ratio of normal volunteers. If the reduction in ACE activity of the family was due to mutation in the C domain active center of somatic ACE, the ratio would have been expected to increase [28]. A ZPHL/HHL ratio for the index patient was not determined since no measurable ACE activity was discernable using either substrate. Furthermore, as revealed in Figure 2, the novel ACE mutation identified (Q1069R substitution) is unlikely to affect the catalytic properties of the C-domain active center and should not directly impact upon the N-domain active center. Analysis of the 3D-structure of the C domain demonstrated that Gln1069 is close to the surface of the C domain and thus far way from the active site making it unlikely that it would have a direct effect on the catalytic activity (Fig. 2).

### Characterization of Q1069R ACE Mutant

There are several possibilities as to why this particular mutation leads to absence of ACE protein in the plasma and presumably the surface of endothelial cells (the main source of circulating ACE). We hypothesized that the absence of plasma ACE could be due to destabilization of mRNA, proteolytic cleavage by intracellular proteases as a result of misfolding of the mutant protein, and impaired trafficking of the mutant to the plasma membrane.

Previously, we showed that dendritic cells (DC) originating from Acute Myeloid Leukemia (AML) blasts has no cell surface ACE, in sharp contrast to DC generated from monocytes of healthy donors [10]. Furthermore, we demonstrated that this was due to impaired trafficking of ACE from the cytosol to cell surface in AML blasts and we were able to restore normal trafficking by treatment with chemical chaperones (sodium butyrate) or cultivation at low temperature (30°C)[Galtseva et al. in preparation].

Several ACE glycosylation mutants that do not have ACE catalytic activity, when transfected into CHO cells (for example a mutation in the N domain in which 4 glycosylation sites are removed, provided by Dr. E. Sturrock, University of Cape Town, South Africa) showed significant ACE catalytic activity after cultivation at lower temperature or in the presence of sodium butyrate-data not shown- personal communications). Therefore, we hypothesized that the new mutation in ACE (Q1069R substitution) caused misfolding of ACE and impaired trafficking of ACE from the endoplasmic reticulum to the cell surface, and thus accumulated inside the cells.

### Site-directed mutagenesis of human recombinant somatic ACE

To decipher the mechanism by which the novel Q1069R mutation might be responsible for the absence of plasma (and perhaps tissue) ACE in the affected individuals, we performed site-directed mutagenesis of human recombinant somatic ACE, generated this mutant of somatic ACE in CHO cells, and compared the biochemical and immunological characteristics of this mutant with WT somatic ACE.

#### Western Blotting

**Figure 3:**
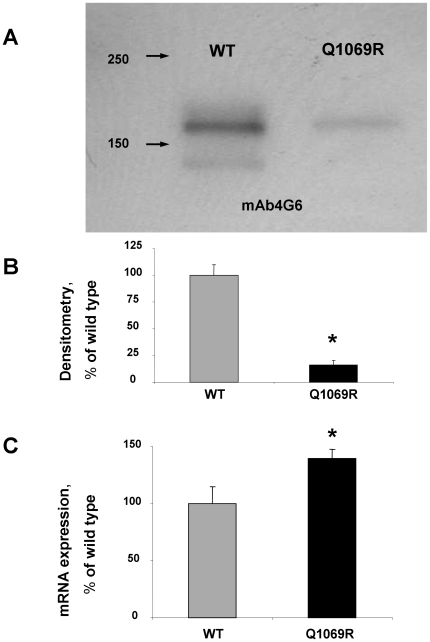
Mutant ACE protein and mRNA quantification. CHO cells were transiently transfected with plasmids coding for wild-type (WT) and mutant (Q1069R) ACE (4 µg of plasmid DNA per 35 mm dish). The lysates of these cells (normalized by equal protein loading of 10 µg per lane) were subjected to SDS-PAGE (4–15% gradient gel) in reducing conditions for Western blotting (**A**) or ACE mRNA quantification (**C**). **A**. Western blotting was performed with rat mAb (4G6) that recognizes the denatured epitope on the N-domain of human ACE [38]. Proteins transferred on PVDF-Plus membrane were revealed with 2 µg/ml of indicated mAb. Molecular weight markers are shown by arrows on the left of panel A, which is a representative experiment. **B**. The relative amount of WT and mutant ACE revealed by Western blotting with mAb 4G6 (**A**) was quantified by the image analysis (densitometry) using ImageJ software (NIH). Data are expressed as mean ± SD of 3 independent experiments. **C**. Relative mRNA concentrations were calculated from the takeoff point of reactions using manufacturer's software and normalized to α-tubulin. Data are expressed as mean ± SD of 3 independent experiments.

For the initial characterization of mutant ACE produced by CHO cells, we performed Western blotting of cell lysates from CHO-WT-ACE and CHO-Q1069R-ACE using several mAbs to denatured human ACE: mouse mAb 1D8, 3C5, and 2E2, which recognize sequential epitopes in the C domain [35]–[37], as well as rat mAb 4G6, that recognizes an epitope on the mouse and human N domain [38]. Fig. 3A showing the Western blot of human ACE using mAb 4G6 demonstrates the classical duplet bands, reflecting the fully glycosylated form of recombinant WT ACE (higher MW band) and the unglycosylated form of recombinant ACE (minor band)[39]. The width and density of the mutant ACE band was much smaller, reflecting a reduced amount of mutant ACE produced by CHO cells from an equivalent number of cells. Similar results were obtained by Western blotting these samples with 3 other mAbs to ACE (not shown). Because the cell lysates were normalized to total cell protein, the dramatic (5-fold) difference in amount of WT and mutant ACE protein (Fig. 3B) might be explained by a reduction in the level of mRNA for mutant ACE (for example due to the effect of this mutation on mRNA stability) or due to intracellular proteolysis of misfolding of mutant ACE.

#### mRNA quantification

In order to determine the reason for the dramatic decrease of mutant ACE protein in transfected CHO cells, we quantified mRNA production by CHO-ACE-WT and the mutant Q1069R. Fig. 3C convincingly demonstrates that c.3293A>G substitution did not decreases the synthesis of mRNA of mutant ACE; rather, the mutant showed even greater ACE mRNA was being produced. Therefore, we should concluded that the dramatic decrease in total mutant ACE protein produced by CHO cells transfected with plasmid coding for mutant (Q1069R) ACE was due to intracellular proteolysis of misfolded mutant ACE.

#### Immunostaining and Confocal Microscopy

**Figure 4:**
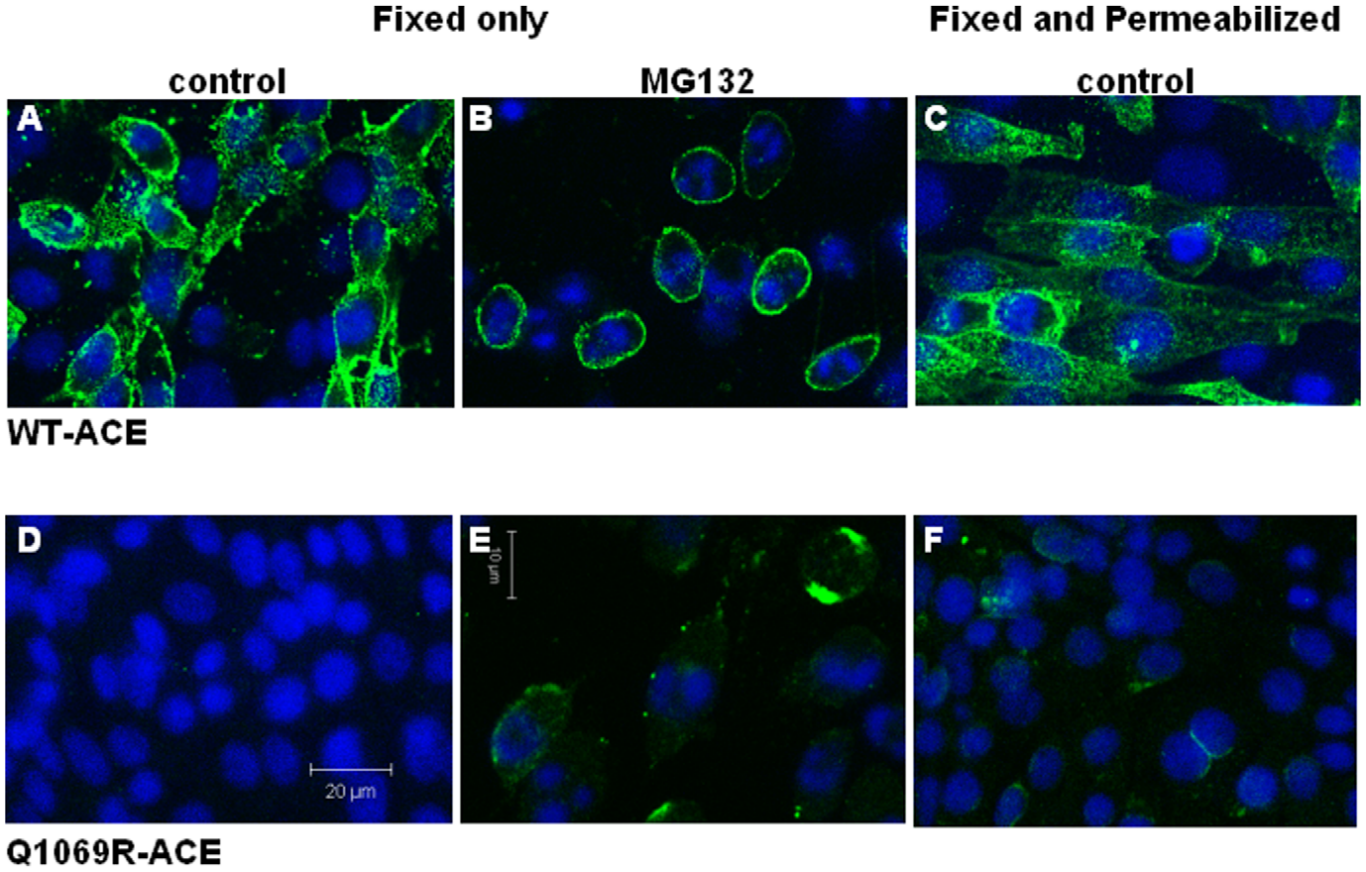
Differential subcellular localization of wild type and mutant ACE. Confocal microscopy was used to determine the localization of WT- vs. Q1069R-ACE mutant expressed in CHO cells following immunostaining with anti-ACE mAb 9B9 and Alexa 488 goat anti-mouse secondary Ab. A-C: WT-ACE expressing cells; D-F: Q1069R-ACE expressing cells. A,B and D,E. Immunostaining of WT and Q1069R ACE in 0.3% paraformaldehye for 15 min at 4C (fixed only). B and E. Treatment with the chemical chaperone sodium butyrate and proteosome inhibitor MG132 for 24 hrs followed by immunostaining with 9B9 mAb. C and F. CHO cells fixed and permeabilized with 3% paraformaldehyde for 15 min at 37C and labeled as above show WT-ACE on the plasma membrane and to a lesser extent in the cytosol and Q1069R-ACE in perinuclear areas.

Confocal microscopy was used to determine the subcellular localization of WT- vs. Q1069R-ACE mutant expressed in CHO cells following immunostaining with anti-ACE mAb 9B9. Fig. 4A shows abundant membrane localization of WT-ACE in lightly fixed-non-permeabilized cells, whereas we did not observe membrane localization of mutant Q1069R-ACE under the same conditions and detector settings (Fig. 4D). To determine whether mutant ACE was expressed, but localized intracellulary, CHO cells were fixed and permeabilized with 3% paraformaldehye for 15 min at 37°C (Fig. 4C and 4F). In WT-ACE expressing cells, ACE was observed primarily on the plasma membrane and to a lesser extent in the cytosol (Fig. 4C), whereas Q1069R-ACE was found mainly in perinuclear areas (Fig. 4F). The perinuclear distribution of mutant ACE is consistent with retention of the protein in the ER and/or Golgi (Fig. 4F). Thus, compared to the non-permeabilized cells, WT-ACE immunostaining remained primarily associated with the plasma membrane whereas Q1069R-ACE immunolocalization was observed typically on one side of the nucleus (Fig. 4F). To clarify whether the trafficking defect of mutant ACE could be rescued and thus restore ACE localization to the plasma membrane, cells were treated with the chemical chaperone sodium butyate and proteasome inhibitor MG132. In WT-ACE expressing cells, treatment with the combination of sodium butyrate acid and MG132 resulted in enhanced ACE immunostaining on the plasma membrane (Fig. 4B). Q1069R-ACE immunostaining was more abundant than in permeabilized cells and it was localized on the membrane following sodium butyrate and MG132 treatment suggesting the pharmacologic agents stimulated trafficking to the surface and also prevented ACE degradation (Fig. 4E). A cytotoxic effect of the drug treatments was also observed (cell rounding and nuclear fragmentation). Therefore, these studies indicate that the Q1069R-ACE mutation impairs trafficking to the cell surface and promotes degradation by intracellular proteases.

During the last 20 years, numerous ACE mutations have been generated (more than 40) and among them, several where identified which had no enzymatic activity but measurable mRNA expression or ACE protein production; these were shown to be due to mutations of potential glycosylation sites. Thus several multiple and single glycosylation mutants were characterized by the arrest of mutant protein in the endoplasmic reticulum and by rapid intracellular degradation [40]–[41] (Sturrock ED, personal communication). Two ACE chimeras were generated by substituting regions of the C domain with corresponding N domain sequences which also demonstrated defective intracellular processing, were enzymatically inactive, and did not localize to the cell surface [42]. A deletion of 4 amino acid residues -Δ9NFSA12 [43], and a single S528C substitution, [Danilov, unpublished observation] that destroys one of the disulfide bridges in the N domain [44] also prevented proper intracellular processing of mutant ACE and lead to fast intracellular degradation. Furthermore, a new human ACE mutation (Δ1141–1152 in the C domain of ACE) found recently in Japan [45] also likely led to the arrest of intracellular processing and rapid degradation. Therefore, it seems that impaired trafficking and fast intracellular degradation as a result of Q1069R ACE mutation is not unique for ACE.

#### ACE activity of the mutant ACE

**Figure 5:**
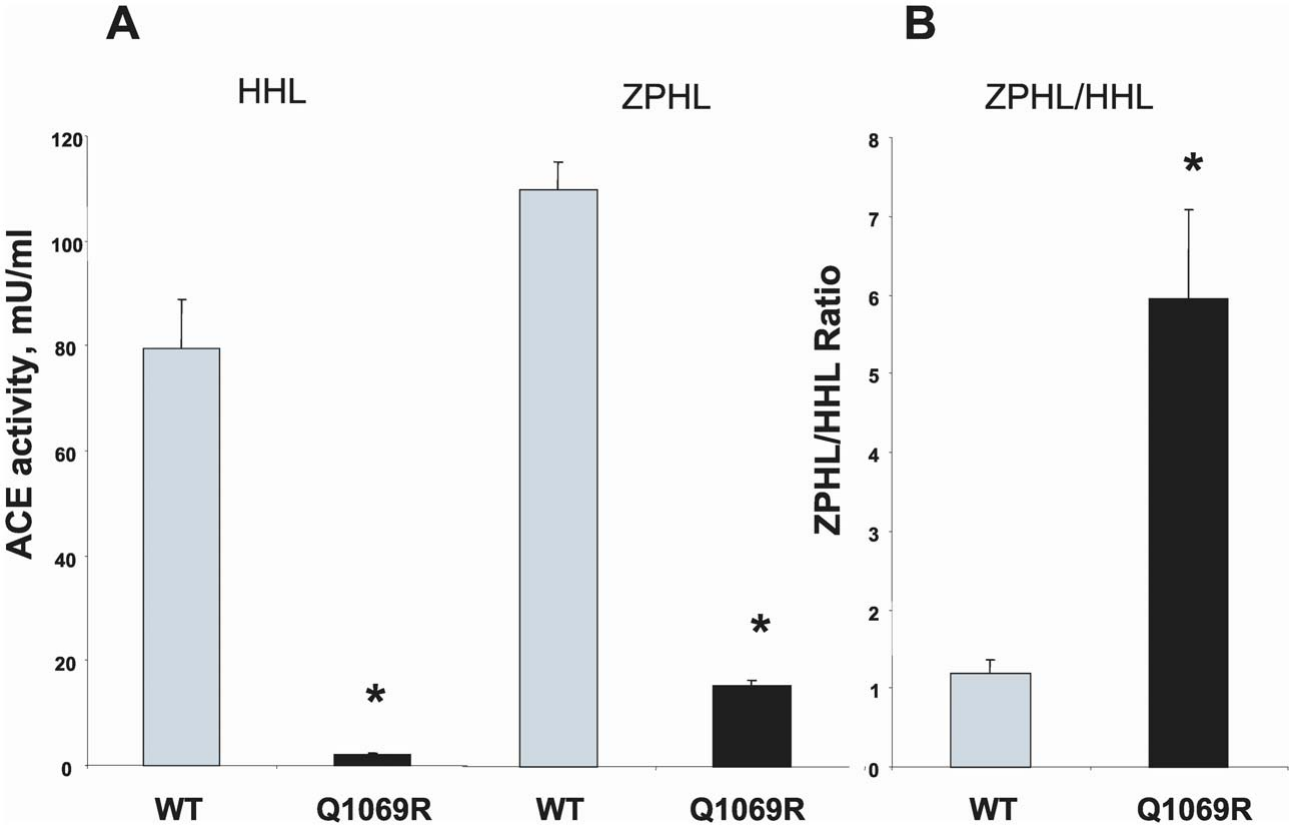
ACE activity of mutant ACE. ACE activity of the membrane-bound form of ACE in lysates of CHO cells expressing WT and mutant ACE was determined by fluorimetric assay of 40 µl aliquotes of cells with 200 µl each of substrates Hip-His-Leu (HHL) and Z-Phe-His-Leu (ZPHL) during 1 hr incubation. **A.** ACE activity with HHL and ZPHL (mU/ml), correspondingly, from a representative experiment performed in triplicate. B.**** Ratio of hydrolysis of the two substrates (ZPHL/HHL ratio) in the tested samples. The data are presented as mean (± SD) from three independent experiments. *p<0.05 vs. control samples.

Kinetic analysis of soluble mutant ACE as well as membrane bound mutant in comparison with WT ACE using two widely used ACE substrates showed a very unexpected result. ACE activity of the lysates of CHO cells expressing mutant human ACE determined with Hip-His-Leu was very low and consisted of about 2.6% of the WT ACE activity in CHO cells (Fig. 5A). This value is significantly lower than the amount of mutant ACE revealed by Western blotting (10–20%; Fig. 3B). However, it was expected, because we and others demonstrated previously that catalytically active ACE localized on the cell surface represents only part of the total ACE produced by cells [6], [46]–[47]. The remaining ACE protein in cells represents immature ACE, perhaps still containing the signal peptide [40], is catalytically inactive [6], [47], perhaps due to improper folding. Western blotting, which was performed with mAbs recognizing denatured epitopes (Fig. 3A), reflects both mature and immature ACE in the cells, with the immature, inactive ACE localized inside cells and mature, catalytically active ACE localized to the cell surface. ACE activity of lysates is thus only a measure of mature, catalytically active ACE on the cell membrane.

ACE activity in cell lysates of CHO-Q1069R-ACE cells determined using the substrate Z-Phe-His-Leu was significantly greater (14.1%) than observed with Hip-His-Leu (2.6%), although still much less than WT ACE (Fig. 5A). Therefore, the ZPHL/HHL ratio for mutant ACE was about 5-fold higher than WT ACE (Fig. 5B). The same difference in this ratio was observed for soluble ACE in the culture fluid (not shown).

The two domains of ACE hydrolyze the same range of natural and synthetic substrates, but with rather different efficiencies [48]–[51]. The two synthetic substrates - Z-Phe-His-Leu (ZPHL) and Hip-His-Leu (HHL) are used for determination of ACE activity in laboratories worldwide, mostly at fixed concentrations, 2 mM and 5 mM, respectively. These two substrates display some contrasting enzymatic properties: the C domain of human ACE hydrolyzes HHL at a much faster rate (9-fold) compared to the N domain [48], whereas ZPHL is hydrolyzed at an almost equal rate by both domains [30]. As a result, the ratio of the rates of hydrolysis of these two substrates (ZPHL/HHL ratio) is characteristic for each type of ACE: somatic (two-domain) human ACE - about 1–1.5, N-domain – 5–7, and C-domain - 0.6–0.8 [28], [52]. We also demonstrated that selective inactivation or inhibition of the C-domain in somatic ACE increases this ratio from 1 toward higher values more characteristic for the N-domain, whereas selective inactivation or inhibition of the N-domain in somatic ACE decreases the ratio toward lower values predicted for C-domain substrate hydrolysis [28].

Data from Fig. 5C indicate that mutant ACE has no functional C-domain active center or that this mutation abolished substrate specificity of the C-domain. The C-terminal domain of WT human ACE cleaves Hip-His-Leu much faster than the N-domain, whereas the N-domain cleaves substrates such as LH-RH and AcSDKP much more efficiently [48]–[51].

We (and others) hypothesized that several amino acid residues might determine a significant substrate specificity of the N- and C-domains. Based on analysis of the rate of hydrolysis of different substrates and the response to different inhibitors and effectors (sulfate ions, dinitrofluorobenzene) by ACE from different species, we hypothesized that the amino acid residues Ser858, Met907, Lys1005, Glu1029, Val1104 in the C-domain might determine preferential hydrolysis of HHL by the C-domain and low efficiency of HHL hydrolysis by the N-domain, giving rise to a high ZPHL/HHL ratio for the truncated N-domain [Danilov, unpublished observation]. Recently, analysis of the 3D structure of the C-domain with novel C-domain selective inhibitors revealed several amino acid residues that may contribute to the domain specificity of ACE inhibition [53]. Interestingly, two residues - Ser858 and Glu1029 (out of 10 suspicious residues), were as we predicted. Furthermore, the structural basis for substrate specificity of the N- and C-domain active centers was partially resolved by systematic mutagenesis of the suspected residues in truncated N- and C-domains. The amino acid residues in the C-domain and N-domain determine (at least in part) the substrate specificity and reason for the low ZPHL/HHL hydrolysis ratio for the C-domain and high ratio for the N-domain (Sturrock ED, personal communications).

**Figure 6:**
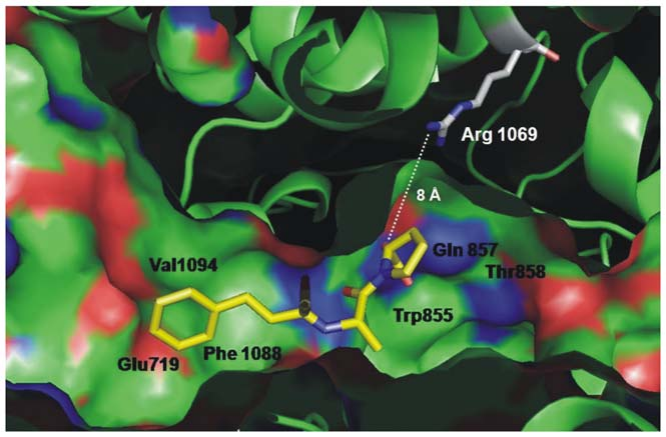
Localization of critical residues in the active center of the C-domain. The localization of amino acid residue Arg1069 relative to the active center of the human ACE C-domain is shown in the ribbon representation of the substrate-bound crystal structure of the C domain fragment in which the 36 amino acid residues unique to tACE have been deleted [75]. Key amino acids referred to in the text crucial for substrate specificity and ACE inhibition are denoted by somatic ACE numbering. Arg1069 is located more than 8 Å from the nearest residue (Pro) of ACE inhibitor enalaprilat shown in yellow.

A closer look to the structure of the active center of the C-domain ruled out the possibility that the Q1069R substitution is responsible for the dramatic increase in ZPHL/HHL ratio of mutant ACE. Figure 6 clearly shows that the distance between Q1069 (or R1069 after substitution) and the proline of the ACE inhibitor lisinopril is about 8 Å, which is too far for a direct interaction with substrates or interference with the residues in the C-domain active center responsible for substrate specificity or domain specificity of ACE inhibition [53]. Therefore, the more likely reason for the apparent non-functional C-domain of mutant Q1069R ACE may be selective denaturation of the C-domain.

#### Immunological characterization of the mutant ACE

We developed a set of mAbs that recognized 16 different conformational epitopes on the surface of the N- and C-domains of human ACE useful for epitope mapping. Eight mAbs recognize epitopes on the catalytically active N-domain [15], [30], [32]–[34] and 8 mAbs recognize epitopes localized on the C-domain of ACE [16], [36]. We recently demonstrated that the pattern of precipitation of ACE activity (conformational fingerprinting of ACE) using this set of mAbs provides a sensitive means of identifying changes in local conformation of ACE due to inactivation, inhibition, mutations, etc. [Danilov et al, 2010, in preparation). Moreover, we successfully used this set of mAbs to detect several new human ACE mutations and characterized the conformation of Pro1199Leu [54] and Trp1197Stop ACE mutations [55].

**Figure 7:**
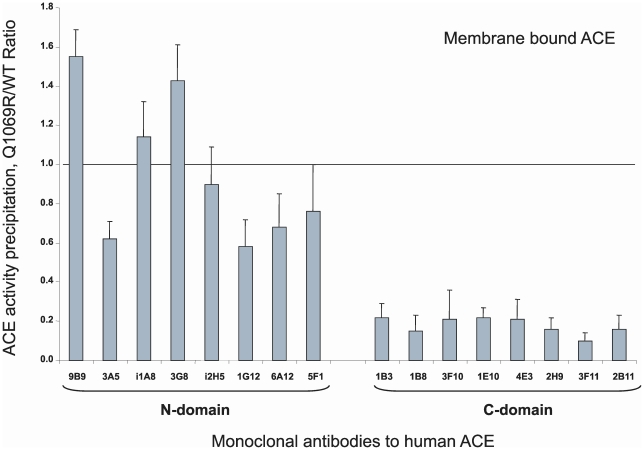
Conformational fingerprinting of mutant ACE. Membrane-bound WT and mutant ACE lysates were normalized to achieve 5 mU/ml ACE activity with Z-Phe-His-Leu as substrate and incubated in microtiter plate wells covered with 16 mAbs to human ACE [16], [30] via goat-anti-mouse IgG. Precipitated ACE activity was quantified by fluorimetric plate precipitation assay [30], [54]. Data (mean ± SD of 6–8 independent experiments in duplicate) are expressed as ratio of ACE activity precipitated by mAbs from mutant ACE to that of WT ACE. * p<0.05 vs. WT ACE.

We thus immunoprecipitated mutant membrane-bound ACE from lysates of CHO cells expressing WT vs. Q1069R ACE (as well as soluble ACE from culture fluids from these cells) to fingerprint the conformation of the mutant. The ability of each mAb to immunoprecipitate ACE is influenced by the local ACE conformation which of course can be influenced by changes of amino acid sequence induced by genetic mutations or by local, selective denaturation. As clearly apparent from Fig. 7, the immunoprecipitation profile of mutant ACE differs dramatically from that of WT ACE. The overall precipitation of ACE activity by mAbs directed to the epitopes localized on the N-domain did not changed dramatically; the Q1069R/WT ratio of ACE activity precipitation for 7 mAbs to N domain calculated from the data presented in Fig. 7 was 1.01±0.38. The ratio for mAb 3A5 was not included in the calculation because this mAb is strongly anti-catalytic and changed significantly the catalytic characteristics of the N-domain of somatic WT ACE [28], [33]. However, the local conformation of certain regions of the N-domain of mutant Q1069R ACE differed significantly. Binding of mAbs to a region of overlapping epitopes for mAbs 9B9, 3G8, and i1A8 [30], [32], [43] visibly increased, whereas binding of mAbs 1G12 and 6A12 whose epitopes also overlap [15], [30] decreased. Precipitation of ACE activity by all mAbs raised against the C-domain was dramatically reduced. Overall, the Q1069R/WT ratio for the 8 mAbs to the C-domain dropped more than 5-fold (0.18±0.04, p<0.05 in comparison with this parameter for mAbs to the N-domain). The same pattern was characteristic for soluble WT and mutant ACE (data not shown). This data unequivocally indicates that a dramatic increase in kinetic parameters of mutant ACE (ZPHL/HHL ratio) was due to selective denaturation of the C-domain of mutant ACE, perhaps due to incorrect chaperoning and more defective trafficking to the cell surface. Moreover, selective denaturation of the C-domain also changed the conformation of regions in the N-domain of ACE.

Increased fragility of the C-domain in comparison with the N-domain was demonstrated first by studying thermal stability of the separated N- and C-domains [56]–[58]. The C-domain was thermally less stable than the N-domain even in native somatic ACE, as demonstrated first by differential scanning colorimetry [57] and later using a kinetic approach utilizing the ratio of the rate of hydrolysis of two substrates for ACE -ZPHL/HHL ratio [28]. The C-domain was also more susceptible to proteolytic degradation than the N-domain [59]–[60]. Therefore, these data provide the basis for suggesting that selective denaturation of the C-domain in mutant ACE which leads to kinetic inactivation of the C-domain active center might be due to the more labile nature of the C-domain as it appears to be less resistant to the effect of chaperones that traffic the misfolded mutant to the cell surface and less resistant to intracellular proteolysis.

**Figure 8:**
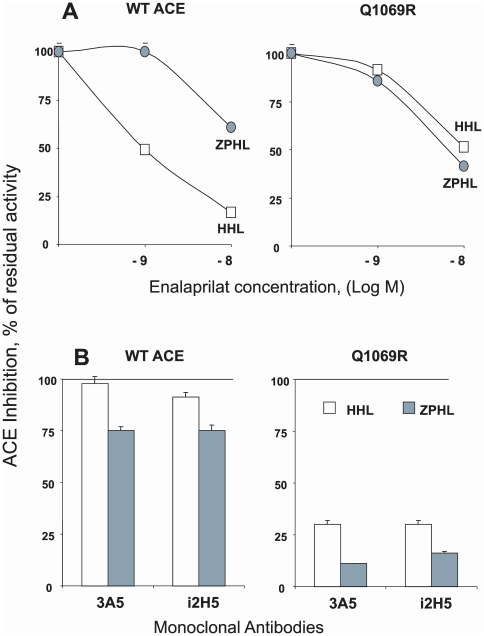
Effect of ACE inhibitors and anti-catalytic mAbs on mutant ACE activity. Lysates form CHO cells expressing mutant and WT membrane-bound ACE were incubated with ACE inhibitor enalaprilat (10^−9^–10^−8^ M) (**A**) and with mAbs (10 µg/ml) (**B**) which are anti-catalytic for the N-domain active center (i2H5 and 3A5- [30], [33]. Residual ACE activity was determined as in Fig. 5.

Selective inactivation of the active center in the C-domain and partial denaturation of this domain was confirmed by analysis of mutant Q1069R ACE inhibition by ACE inhibitor enalaprilat and by anti-N-domain catalytic mAbs 3A5 and i2H5 (Fig. 8). Inhibition of WT ACE mediated Hip-His-Leu hydrolysis was much more profound than inhibition of ZPHL hydrolysis (Fig. 8A). This might be explained by the preferential inhibition of the C-domain active center in native ACE by commercial ACE inhibitors [28], [61] and by preferential hydrolysis of HHL by the C-domain active center [48], whereas the anti-catalytic activity of enalaprilat on mutant ACE (which contains only the N-domain active center) was similar with both substrates (which corroborates well with the fact that the rate of hydrolysis of these two substrates by the N-domain active center is similar [30]. Inhibition of ACE activity (HHL cleavage) in WT ACE by anti-catalytic mAbs towards N domain was practically absent due to this reason, whereas their action on ZPHL hydrolysis was visible, but did not reach 50% inhibition at the concentration of mAbs used because these mAbs inhibited only the N domain center and hydrolysis of the substrates in the C-domain was not disturbed. The anti-catalytic affect of these mAbs was quit profound towards mutant ACE, confirming that the hydrolysis of the substrates by mutant ACE was performed mainly by the N-domain active center.

**Figure 9:**
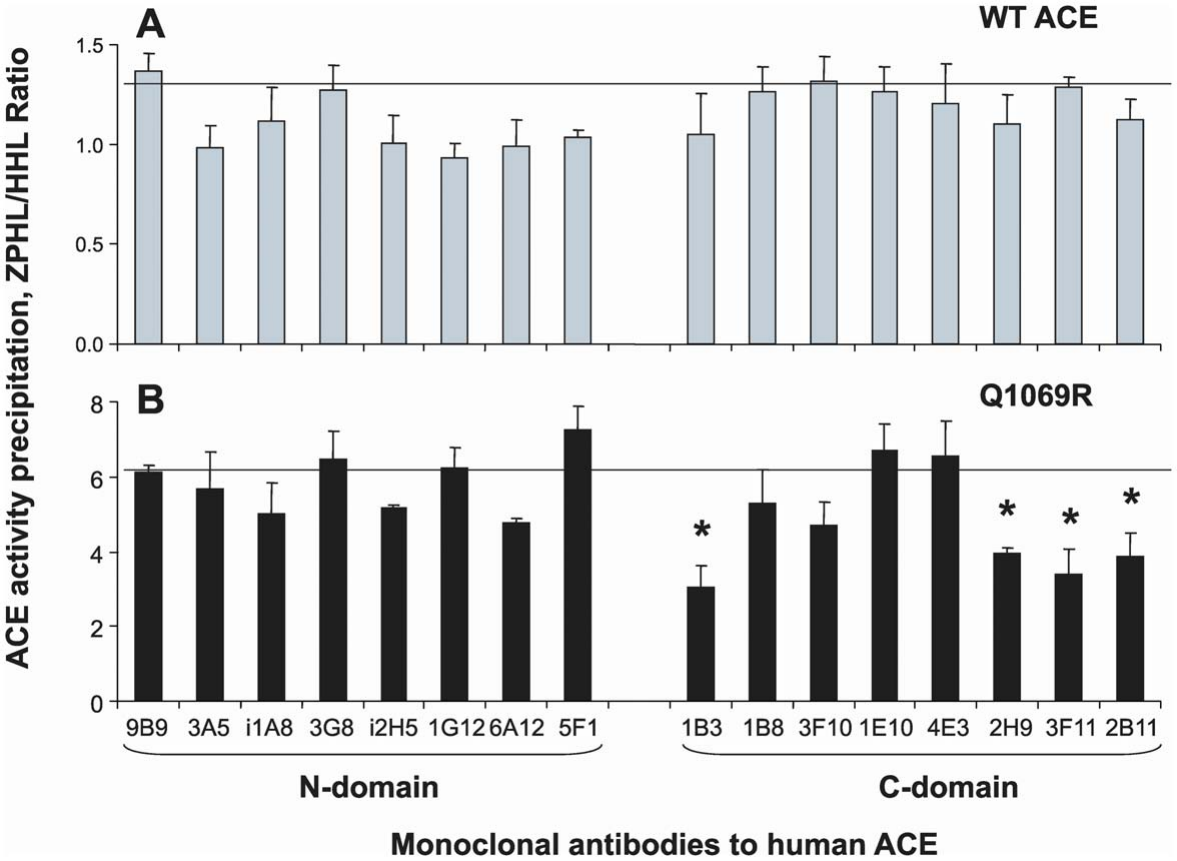
Catalytic properties of mutant ACE bound to ACE mAbs. Membrane-bound WT (**A**) and mutant ACE (**B**) lysates were normalized to achieve 5 mU/ml ACE activity with Z-Phe-His-Leu as substrate and incubated in microtiter plates covered by 16 mAbs to human ACE as in Fig. 7. ACE activity precipitated by each mAb was quantified by fluorimetric assay with two substrates (Hip-His-Leu and Z-Phe-His-Leu) as in Fig. 5. Data are expressed as the ratio of ACE activity precipitated by each mAb determined with each substrate. Data are mean ± SD of 6–8 independent experiments in duplicate. * - p<0.05 vs. ZPHL/HHL ratio for corresponding values of WT and mutant ACE in solution (horizontal lines in A and B). The ratio for duplicate samples of WT ACE or mutant ACE was approximately 1.0 and the SD was less than 10%.

We also assessed ZPHL/HHL hydrolysis by mutant ACE bound to antibodies. Fig. 9 demonstrates that the ZPHL/HHL ratio of WT ACE did not significantly change relative to WT ACE in solution (horizontal line in Fig. 9A). However, selective denaturation of the C domain of mutant ACE (as demonstrated in Fig. 7) was partially restored after binding to some of the mAbs. The ZPHL/HHL ratio for the mutant ACE precipitated by mAbs to the N-domain was the same as for mutant ACE in solution (horizontal line at Fig. 9B), whereas the ZPHL/HHL ratio for mutant ACE trapped by some mAbs to the C-domain significantly decreased, demonstrating a movement toward lower values characteristic of native two-domain ACE (Fig. 7B). mAb is 1B3 directed to an epitope close to the C-terminal end of the C-domain, whereas mAbs 2H9, 3F11 and 2B11 recognize highly overlapping epitopes on the other side of C-domain globule [16].

**Figure 10:**
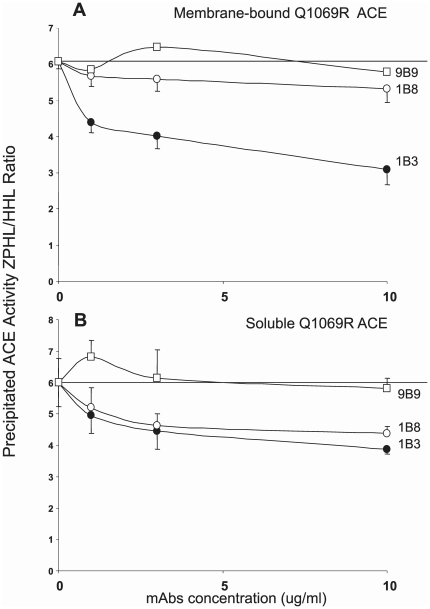
Chaperone-like effect of anti-ACE mAbs on mutant ACE. Membrane-bound (**A**) and soluble (**B**) mutant ACE (Q1069R) were pre-incubated with N-domain specific mAb 9B9 [30] or C-domain specific mAbs 1B3 and 1B8 [16], [36] at the indicated concentrations for 2 hrs at room temperature and then residual activity was determined with 5 mM HHL and 2 mM ZPHL as substrates. Experimental conditions are as described in the legend to Fig. 5. Data presented as ZPHL/HHL ratio of ACE activity in the presence mAb. Results shown are the mean ± SD of 3–4 experiments.

We confirmed that this chaperone-like effect of these mAbs to ACE was also retained when mutant ACE was in solution as in the situation described in Fig. 8. Fig. 10 demonstrates a dose-dependent decrease of ZPHL/HHL ratio for mutant ACE indicative of partial renaturation after adding mAb 1B3. mAb 9B9, whose epitope is localized on the N-domain served as a negative control. It is interesting to note that the chaperone-like effect of mAb 1B3 was similar for membrane-bound mutant ACE (Fig. 10A) and for soluble mutant ACE (Fig. 10B). This effect of mAb 1B8 was very modest for membrane-bound ACE (Fig. 10A) and more profound in the case of soluble ACE (Fig. 10B), which is consistent with the fact that the epitope for mAb 1B8 is more exposed on soluble ACE in comparison with membrane-bound ACE [16].

A chaperone-like effect of the mAbs and the partial or complete renaturation of *reversibly* denatured protein conformations were previously demonstrated for other antigens [62]–[64]. Chaperone-like antibody activity may be due to stabilization of native antigen conformations, folding transition states, or screening of aggregating hydrophobic surfaces [63].

### Pharmacological rescue of conformation-defective ACE mutant

There are now hundreds if not a thousands of examples of transport-defective mutations to proteins. Mutations resulting in the changes in protein sequences often result in the production of misfolded and disease-causing proteins that are transcribed and translated at normal levels but are unable to reach their functional destination in cells (for review see Sanders and Nagy, [65]. Misfolding results in loss-of-function of the conformationally defective protein via degradation through the polyubiquitination/proteasome pathway [66]. One of the best described examples of a transport-defective mutation is the deletion of Phe508 in the cystic fibrosis transmembrane conductance regulator (Δ F508 CFTR). The defective protein retains substantial chloride-channel function in cell-free lipid membranes, but when synthesized, the protein is rapidly recognized as misfolded and is degraded shortly after synthesis before it can reach its crucial site of action at the cell surface [67]. Alternatively, misfolded proteins may aggregate, leading to potentially toxic intracellular accumulation of even to protein accumulation in plasma with extracellular amyloid deposits. Well described examples of such scenario's are observed in neurodegenerative disorders such as Alzheimer's Disease, Parkinson's Disease, Huntington Syndrome, and prion and motor neuron disorders [68].

In the past decade, extraordinary efforts have been made to understand how abnormal folding relates to certain pathologies and to design therapeutic interventions that prevent or correct the structural abnormality of the disease-causing misfolded proteins. In this regard, rescue of misfolded “trafficking-defective” proteins by chemical and pharmacological chaperones and by inhibitors of intracellular degradation is emerging as one of the most promising therapeutic strategies for such disorders [69]–[71].

The clarification of the molecular mechanism of this particular ACE mutation has not only scientific, but also clinical value. If the treatment of CHO cells expressing mutant ACE with a combination of chemical and pharmacological chaperones for ACE and proteasome inhibitors can effectively restore ACE trafficking to the cell surface *in vitro*, it would be logical to suggest that these treatments may also increase ACE trafficking and thereby prevent fast intracellular proteolysis in patients. We hypothesize herein that ACE inhibitors might also be considered as pharmacological chaperones for ACE.

**Figure 11:**
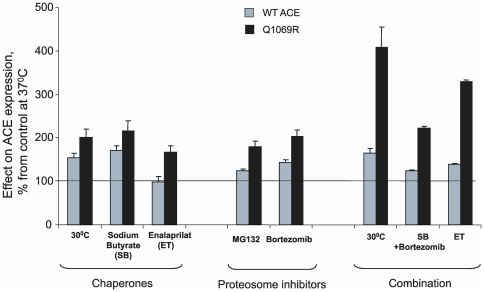
Effect of temperature, chaperones, and proteolytic inhibitors on ACE activity. ACE activity of membrane-bound form of WT and mutant ACE after culturing cells 24 hrs in different conditions listed below as described in Materials and Methods. ACE activity was determined by fluorimetric assay as described in the legend to Figure5 using Z-Phe-His-Leu (ZPHL) as substrate. The following compounds were tested: Sodium butyrate (5 mM); Enalaprilat (1 µM), MG132 (5 µM), Bortezomib (5 µM). Data are the mean values (± SD) of 3–4 independent experiments measured in duplicate expressed as the percentage of ACE activity in the lysate of WT or mutant ACE expressing cells cultured at 37°C.

In order to improve the trafficking of mutant ACE to the cell surface, we cultivated CHO cells expressing mutant and WT ACE in various conditions known to increase trafficking of proteins to the cell surface and prevent intracellular degradation. Fig. 11 demonstrates that culturing at low (30°C) temperature or with addition of chemical chaperone (sodium butyrate), pharmacological chaperone (ACE inhibitor), or proteasome inhibitor (MG132 or bortezomib) alone or in combination dramatically increased cell surface expression of mutant ACE. ACE activity in lysates of CHO cells expressing mutant ACE significantly increased as a result of such an intervention and reached approximately 60% of that of WT ACE. A significant effect of these compounds or low temperature was also observed on the trafficking of WT ACE, though the magnitude of the changes was less than that observed with mutant ACE. A similar response to treatment with chemical chaperones rescued the trafficking of mutant bone morphogenetic protein receptor II (BMPR-II), and thus impaired trafficking was demonstrated to be responsible for the development of pulmonary hypertension [72].

We should note that a combination of similar treatments, for example low temperature and sodium butyrate, did not show an additive effect (data not shown), whereas the combination of low temperature with the proteasome inhibitor bortezomib had a profound additive effect (Fig. 11). Therefore, we believe that selective combination of chaperones and proteasome inhibitors may restore (at least partially) impaired trafficking of mutant ACE to the cell surface in patients.

Based on these results (Fig. 11) and the fact that the parents of this patient have approximately two-times less ACE compared to the population norm (Fig. 1), such an approach might work for patients with this mutation (Q1069R) and perhaps others, such as the patient in Japan with the Δ1141–1152 ACE deletion in the C-domain [45].

The complete absence of ACE during fetal development caused structural malformations of the renal tubular system of the index patient. Thus, it is possible that restoration of impaired ACE trafficking to the surface of ACE expressing cells after treatment with chemical or pharmacological chaperones and the generation of AII by renal proximal tubule epithelial cells may not improve clinical symptoms in this patient. However, there are at least two arguments based on mouse models that did not develop anuria but had urine concentrating defects that support the hypothesis and goal of improving clinical symptoms in this patient.

First, the ACE.2 strain of mice developed by the K. Bernstein lab [73] show an unusual pattern of RNA splicing resulting in animals with approximately 1/3 normal plasma ACE activity. However, these mice completely lacked tissue ACE activity. The major difference between the ACE.2 mice and ACE null mice completely lacking ACE activity was the renal pathology. The ACE.2 mice presented with a much milder form of the renal medullary and papillary underdevelopment. In the majority of these animals, renal medullary development was approximately equivalent to that of WT mice. However, despite relatively normal renal architecture, the ACE.2 mice were unable to effectively concentrate urine [73]. When this team correlated the ability to concentrate urine with renal pathology among individual animals, they observed examples of mice with perfectly normal renal medullas that were functionally equivalent to the ACE null mice in their inability to concentrate urine. These results imply that the ability to generate angiotensin II locally within the kidney plays a critical role in the ability to properly concentrate urine.

The second argument comes from studies demonstrating that administration of exogenous angiotensin II caused a restoration of tubuloglomerular feedback (TGF) responsiveness to 71% of control in heterozygous and to 62% of control in homozygous ACE null mice. These data support previous conclusions that angiotensin II is an essential component in the signal transmission pathway that links the *macula densa* with the glomerular vascular pole [74].

Thus, we identified the molecular mechanism by which a novel mutation of ACE in which codon CAG coding for Gln at position 1069 is substituted in both alleles by codon CGG for Arg results in nearly complete arrest of intracellular processing and trafficking of ACE to the cell surface. Due to this arrested, the misfolded mutant ACE becomes much more susceptible to proteolysis, undergoes rapid intracellular degradation, and does not traffic to the surface of ACE expressing cells like endothelial cells of capillaries or epithelial cells of the kidney. As a result, development of the proximal tubules was impaired and the patient developed renal tubular dysgenesis.

We characterized the molecular basis of defective ACE processing due to this mutation using a cellular CHO expression model in which mutant ACE (Q1069R) activity, immunoreactivity, and trafficking could be defined. Using a novel conformational fingerprinting approach for ACE, we demonstrated that mutant ACE was selectively denatured. The C-domain of somatic ACE was practically non-functional and only the N-domain active center was able to cleave ACE substrates. Moreover, using this model we were able to prevent, to some extent, intracellular degradation of misfolded mutant ACE, and, to rescue, to some degree, trafficking of mutant ACE to the cell surface.

Therefore, this study provides important insights into the mechanism by which the novel ACE mutation (Q1069R) results in dysfunctional membrane ACE expression due to a trafficking defect as well as severe renal tubular dysgenesis. Moreover, we demonstrated an *in vitro* approach to partially rescue impaired trafficking of mutant ACE to the cell surface. It may be that such an approach might result in attenuation of clinical symptoms of patients with this or similar mutations.
